# First TILLING Platform in *Cucurbita pepo*: A New Mutant Resource for Gene Function and Crop Improvement

**DOI:** 10.1371/journal.pone.0112743

**Published:** 2014-11-11

**Authors:** Nelly Vicente-Dólera, Christelle Troadec, Manuel Moya, Mercedes del Río-Celestino, Teresa Pomares-Viciana, Abdelhafid Bendahmane, Belén Picó, Belén Román, Pedro Gómez

**Affiliations:** 1 IFAPA Centro La Mojonera, Camino de San Nicolás, 1, 04745 La Mojonera, Almería, Spain; 2 INRA-URGV, UMR1165, Unité de Recherche en Génomique Végétale, Saclay Plant Sciences, Evry, France; 3 Institute for the Conservation and Breeding of Agricultural Biodiversity (COMAV-UPV), Universitat Politécnica de Valencia, Camino de Vera s/n, 46022 Valencia, Spain; 4 IFAPA Centro Alameda del Obispo, Avd. Menéndez Pidal s/n, 14004, Córdoba, Spain; USDA-ARS, United States of America

## Abstract

Although the availability of genetic and genomic resources for *Cucurbita pepo* has increased significantly, functional genomic resources are still limited for this crop. In this direction, we have developed a high throughput reverse genetic tool: the first TILLING (Targeting Induced Local Lesions IN Genomes) resource for this species. Additionally, we have used this resource to demonstrate that the previous EMS mutant population we developed has the highest mutation density compared with other cucurbits mutant populations. The overall mutation density in this first *C. pepo* TILLING platform was estimated to be 1/133 Kb by screening five additional genes. In total, 58 mutations confirmed by sequencing were identified in the five targeted genes, thirteen of which were predicted to have an impact on the function of the protein. The genotype/phenotype correlation was studied in a peroxidase gene, revealing that the phenotype of seedling homozygous for one of the isolated mutant alleles was albino. These results indicate that the TILLING approach in this species was successful at providing new mutations and can address the major challenge of linking sequence information to biological function and also the identification of novel variation for crop breeding.

## Introduction

*Cucurbita pepo* is the main species of the genus *Cucurbita* and represents one of the most important vegetable crops worldwide in terms of food consumption. Of the eight existing morphotypes in this species, the morphotype Zucchini is the most economically valuable. It was recently developed and displays low levels of genetic variation [1] but nevertheless it is commonly cultivated. Nowadays, in this species the development of new cultivars is based on the availability of variable germplasm. However, apart from the intraspecific resources, grouped in three subespecies, only a few crossable *Cucurbita* species can be used as sources of agriculturally useful alleles and new genotypes. In this way, the development of new genetic resources along with the application of new molecular techniques in breeding would facilitate the improvement of characters, mainly those with more complex regulation, allowing breeders to face new challenges in this species.

The high competitiveness of plant breeding has increased the interest in the development of new sources of genetic variation using reverse genetics approaches. The silencing or interruption of individual target genes provide the opportunity to investigate gene function, linking sequence variation to specific phenotypes [2], [3]. Thus, TILLING (Targeting Induced Local Lesions IN Genomes), which combines traditional chemical mutagenesis with high-throughput genome-wide screening for point mutations in desired genes, has been developed in response. In a TILLING project, chemical mutagenesis based on an alkylating agent like EMS (ethyl methane sulphonate) [4] that causes random point mutations at high density, provides an easy and cost-effective way to establish a series of allelic mutations in the whole gene set of any species. The screen of the mutant population with an efficient method to identify point mutations, for example a mismatch specific digestion followed by a Li-COR separation of the resulting fragments, may allow the identification of multiple alleles for a specific gene regardless of the gene size [5], [6]. If the mutation frequency is high enough most, if not all genes, will present mutated alleles in the population.

This method may be preferable to other reverse genetic approaches for various reasons, for example because EMS generates a large spectrum of mutations, including missense and truncation mutations, allowing more flexibility than insertional mutagenesis or transgenesis [5]. The newly identified alleles in TILLING could be used for elucidating gene function. This strategy is invaluable because it generates allelic series often including null alleles, although at low frequency [7]. TILLING also finds application in crop improvement, as the identified mutations can be readily utilized in traditional breeding programs since it is nontransgenic and the novel variations are stably inherited [8]–[10].

However, to date there are no TILLING-derived crop varieties that have yet been released though there have been many successful examples of TILLING approaches in basic plant science. It has proved to be useful in a large number of agronomically important crops [11] and has allowed to select mutants with allelic variants in virus resistance genes [12], [13], or in genes involved in abiotic stress [14], nutritional properties [15] or shelf life in *Cucumis melo* [16]. Also, some agricultural biotechnology companies have directed this technology towards the enhancement of the tomato shelf life and the reduction of gluten content of wheat for celiacs [2].

Although this reverse genetic approach has been reported in many species, regarding the *Cucurbitaceae* family, only several reports have been published on *Cucumis melo* [13], [16] and recently on *Cucumis sativus* [17]. No TILLING platforms have been reported for other cucurbits species such as *Cucurbita pepo*. The development of a TILLING platform for this species combined with the recent sequence of the genome (a first version available at cucurbigene.net) and other genomic tools such as the first SNP-based genetic map and the first transcriptome [18], [19] could be of great interest, because it could be a way to induce new genetic variation that complements the natural extant variation in this species.

Based on our previously reported method to obtain mutants in *C. pepo* that originated a reference EMS mutant population, with the present study we have established the first TILLING platform developed for this species which could represent an important advance for crop improvement in Zucchini.

## Materials and Methods

### Mutagenized plant material and growing conditions

Field studies were carried out at the IFAPA research center in Almeria (Spain) (GPS coordinates: 36.78881667, −2.703297222) and no specific permissions were required for these locations/activities. The field studies did not involve endangered or protected species.

Four to nine seeds of each of the 1464 M2 families from a mutant population previously generated with three different EMS doses: 80 mM, 65 mM and 40 mM [20] were sown and grown in greenhouses following standard local commercial practices for both plant nutrition and pest and disease control. Germination and sterility rates of the M2 families were evaluated and controlled self-pollinations were attempted on each plant. Female flowers were protected the day before anthesis to prevent the transfer of pollen by insects, and early in the morning each plant was self-pollinated by hand. The plants of each family were self-pollinated until one fruit with seeds per each M2 family was obtained. At 60–80 days after self-pollination, M3 seeds were harvested, aired and stored at 4°C. Phenotypic alterations in M2 plants also were regularly monitored and compared with untreated plants that were grown at the same time.

### Genomic DNA extraction and pooling

Leaf material from only one plant of each M2 family was collected for DNA isolation. The plant was selected if it produced at least 30 viable seeds. Three leaf discs (diameter 10 mm) were collected and DNA was extracted according to Martin et al. 2009 [21]. Genomic DNAs were quantified on 1% agarose gel using λ DNA (Promega Biotech Iberica) as a concentration reference. DNA concentration was normalized to 3 ng/µl and pooled eightfold in a 96-well format. M3 and M2 seeds of all pooled M2 families are maintained at the genebank of the IFAPA research center.

### PCR amplification, mutation detection and validation

Primer sets were designed for five genes (Table S1) and DNA amplification was based on nested-PCR and universal primers to improve the specificity of the amplification [22], [23]. Carotenoid gene structure was established based on the first version of the *C. pepo*, morphotype Zucchini, genomic sequence (available at http://cucurbigene.net) while peroxidase gene structure was derived from NCBI databases. Sequences of the ethylene receptors (CpETR1 and CpERS1) were previously determined in our laboratory from the homology of other cucurbits. Primers were designed to amplify ∼1 kb segments based on the selected *C. pepo* genomic sequences. The first PCR amplification was a standard PCR reaction performed in a 25 µl volume consisting of dH2O, 10× PCR buffer, 2.5 mM MgCl2, 5 mM dNTPs, 1 U Taq polymerase, 10 mM forward and reverse target-specific primers and 4.5 ng of genomic DNA. The thermocycling conditions were 95°C for five minutes for initial denaturing, followed by 35 cycles of 94°C for 15 seconds, 60°C for 20 seconds, 72°C from one to two minutes and one cycle of 72°C for five minutes.

One microliter diluted ten times of the first PCR product was employed for the second PCR with a combination of specific primers carrying M13 tail and M13 universal primers, M13F700 (5′-CACGACGTTGTAAAACGAC-3′) and M13R800 (5′-GGATAACATTTCACACAGG-3′), labelled at the 5′end with infra-red dyes IRD700 and IRD800 (LI-COR, Lincoln, Nebraska, USA) respectively. This PCR was carried out using 0.125 to 0.25 µM of each primer [23], using the following two step cycling program: 94°C for 5 minutes, 10 cycles at 94°C for 15 seconds, primer-specific annealing temperature for 30 seconds and 72°C for 2 minutes, followed by 25 cycles at 94°C for 15 seconds, 50°C for 30 seconds and 72°C for 1 minute, then a final extension of 5 minutes at 72°C. PCR products were separated in 2% agarose gels.

Mutations were detected in the amplified targets using the mismatch-specific endonuclease ENDO1 as previously described Triques et al. 2007 [24]. Electrophoresis was performed on a LICOR 4300 (LI-COR, Lincoln, NE, USA) and gel images were analysed using Adobe Photoshop software (Adobe Systems Inc., San José, CA, USA). After discovery, mutations were validated by sequence analysis. The mutation frequency for each amplicon was calculated as previously described by Dalmais et al. 2008 [23].

### Sequence Analysis Tools

The PARSESNP software (Project Aligned Related Sequences and Evaluate SNPs) [25] was used to illustrate the distribution of mutations within the gene and to indicate the nature of each single mutation. The SIFT software (Sorting Intolerant from Tolerant, http://sift.bii.a-star.edu.sg/) [26], was used to predict the impact of the mutation on the protein. Scores below 0.05 are predicted to affect protein function. Multiple sequence alignment of full-length protein sequences was performed with Clustal Omega software (https://www.ebi.ac.uk/Tools/msa/clustalo/)

### Mutant protein structure modelling and genotype determination

The APRX three-dimensional structure was generated using the Geno3D server (http://geno3d-pbil.ibcp.fr). Superposition of the wild type APRX structure and the mutant APRX protein was carried out and visualized using Chimera (http://www.cgl.ucsf.edu/chimera).

For each APRX mutant line, homozygous and heterozygous M3 plants were determined by sequencing APRX gene.

### How to access the TILLING population

Screening of the *C. pepo* TILLING platform is not established as a service yet but we are currently working on developing a website. For further information about how to access to the platform please contact to: pedro.gomez.j@juntadeandalucia.es

## Results

### Validation of the *Cucurbita pepo* TILLING platform material

A population of 1464 M2 lines from a previous mutagenesis experiment performed with three different EMS dosages (80 mM, 65 mM and 40 mM) was used and the effect on seed germination, plant development and M3 seed production was evaluated.

All untreated plants used as controls germinated. Nevertheless, germination rate was poor in M2 families obtained with 80 mM and 65 mM dosages (52.6%, and 56.37% respectively) and significantly better in families obtained with 40 mM (70.83%). Additionally, growth of M2 plants was delayed and took longer to germinate at the two higher doses. Moreover, increased EMS concentration led to an increased percentage of M2 fruits in which seeds were non-viable or in which the number of viable seeds was too low to perform TILLING analysis, 63% and 56% for the treatment with 80 mM and 65 mM respectively and 36% for 40 mM treatment. In total 95 and 52 M3 families were collected from the 80 and 65 mM treatments, respectively, and 621 M3 families from the 40 mM treatment (Table 1).

**Table 1:** Summary of the *Cucurbita pepo* mutant collection development.

EMS concentration	Obtained M1 plants	M2 seed families	Fruits with less than 30 seeds (%)	M3 seed families
80 mM	4500	259	63	95
65 mM	2700	231	56	52
40 mM	2900	974	36	621

**Figure 1:**
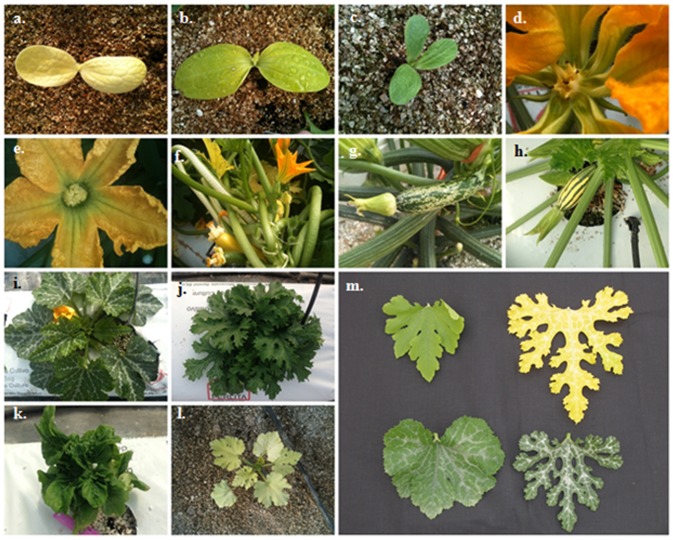
Examples of morphological mutants observed within the *C. pepo* TILLING population. (a) Plant affected in cotyledon colour, albino. (b) Plant affected in cotyledon colour, chlorotic. (c) Plant affected in cotyledon number. (d–e) Female flowers with abnormal stigma. (f–h) Different coloured fruits. (i–k) Semi-dwarf plants with bushy and hyper compact architecture. (l) Albino dwarf plant. (m) Different size and colour leaves.

Compared to untreated plants, each family was evaluated for visual mutations in the phenotypic analysis and a range of different phenotypes was observed in all M2 plants. Albinism and chlorophyll deficiency occurred in 1.3% of the M2 families. About 1.84% showed alterations in cotyledons with variation in their number or shape and 2.2% of the M2 plants segregated individuals displaying “dwarf” or “semidwarf” phenotypes. At flowering and fruit stages the most commonly observed phenotypes were related to the leaf colour and morphology, the growing habit, the plant size or fruit traits. In Figure 1 examples of *C. pepo* mutant phenotypes at key developmental stages are shown confirming the high level of the mutagenesis.

### Identification of mutations in target genes by TILLING: mutation efficiency

To set up the *C. pepo* TILLING platform, DNA samples were prepared from 768 M2 lines, each representing an independent family and organized in pools of 8 M2 families. The selection of M2 families was based on the M3 seed disposability.

To validate the TILLING method and to estimate the mutation density of the population, a set of five genes of *C. pepo* involved in diverse metabolic routes were chosen. Two genes were chosen from the ethylene signaling pathway, in particular of the ethylene response such as ERS1 (ethylene response sensor 1) and ETR1 (ethylene receptor 1). LCYb (lycopene beta cyclase) and PSY (phytoene synthase) were also chosen as important enzymes involved in the carotenoid biosynthesis pathway [27]. The last one was the APRX gene, a gene encoding a peroxidase and that seems to have an important function in the growth and development of the plant [28].

**Figure 2:**
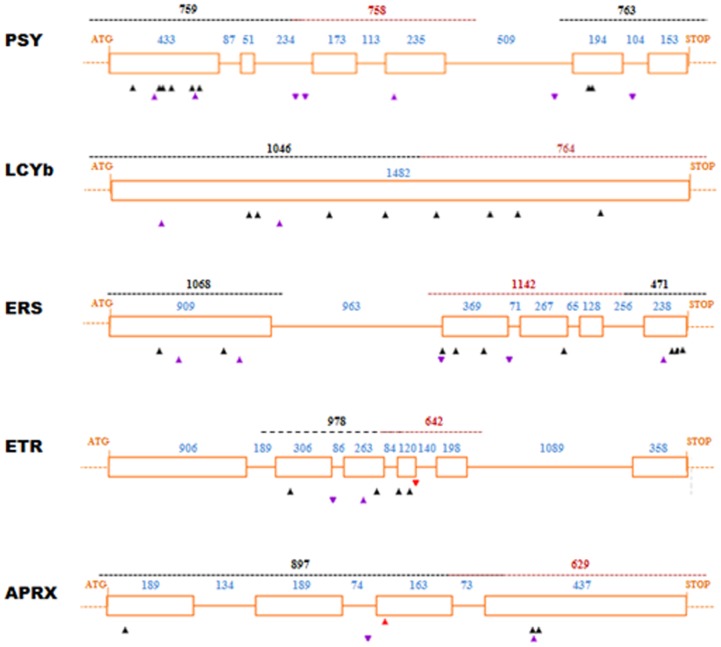
Gene structure of the target genes screened in the *C. pepo* population (PSY, LCYb, ETR1, ERS1, and APRX). This drawing was made using the PARSESNP program, which maps the mutation on a gene model to illustrate the distribution of mutations. Orange boxes represent exons and orange lines introns, the size (pb) is indicated in blue. Dashed lines in red and black indicate amplicons analyzed by TILLING. Purple triangles represent silent mutations and black and red triangles represent missense and truncation mutations, respectively. Mutations before ATG and after STOP codon are not shown.

PCR products used for TILLING have a maximum size of about 1200 bp and, therefore, longer genes were divided into several amplicons. We focused our efforts on identifying for each candidate gene the most promising regions for TILLING analysis. In this way, new primer pairs flanking this region could be targeted to the intronic sequences, with the aim of improving the screening efficiency on the coding regions in the pilot assay (Figure 2). In total 10 Mb of *C. pepo* genomic sequence were screened in 768 M2 plants and 58 induced point mutations were identified of which four and eight mutations were derived from the 65 mM and 80 mM EMS treatment respectively, and 46 mutations from the 40 mM treatment population (Table 2).

**Table 2:** Effect of EMS dose on mutation frequency in *C. pepo* mutant collection.

EMS dose	M2 plants	Induced mutations*	Mutation frequency
80 mM	95	8	1/119 Kb
65 mM	52	4	1/130 Kb
40 mM	621	46	1/135 Kb
**TOTAL**	**768**	**58**	**1/133 Kb**

*Number of identified mutations in all the families for this EMS dose after screening five genes.

Among the 58 identified mutations, we have identified 59% non-synonymous mutations (including nonsense and splicing mutations). Extrapolating from this small set of genes, we calculated an average mutation rate of one in around 133 kb or 6300 mutations per genome for a genome size of 845 Mb [29].

We identified 57 lines, corresponding to 58 different mutations, among which eight missense mutations cause changes in the PSY amino acid sequence, seven in the LCYb amino acid sequence, four and nine in the ETR1 and ERS1 amino acid sequence and four in the APRX amino acid sequence (Table 3). The exonic mutations were mostly present as heterozygotes (36 out of 45 mutations), but 9 lines were homozygous for the mutations. As expected with EMS mutagenesis, these mutations were distributed relatively evenly within the screened amplicons (Figure 2).

**Table 3:** Tilled genes and mutation frequency in the *C. pepo* mutant collection with 768 M2 families screened.

Target names	Amplicon size (bp)	GC content (%)	Identified mutants				
			Missense^a^	Nonsense^b^	Splicing^c^	Silent^d^	Non coding^e^
PSY	2263	41	8	0	0	3	5
ERS1	2663	42	9	0	0	3	4
ETR1	1620	39	4	0	1	1	1
APRX	1398	47	4	1	0	2	2
LCYb	2035	42	7	0	0	2	1
**TOTAL**	**9979**	**42**	**32**	**1**	**1**	**11**	**13**

a nucleic acid transition is a non-synonymous mutation and induce amino acid change in the translated protein.

b nucleic acid transition produces a stop codon and may induce a truncated protein.

c nucleic acid transition is located in splicing motif.

d nucleic acid transition induces a synonymous mutation and then no change in the translated protein.

e nucleid acid transition is located in an intronic or promotor region.

The exonic mutations were mostly present as heterozygotes (36 out of 45 mutations) and 9 lines were homozygous for the mutations. Thus, even though these results are higher than expected, the deviation between data is not statistically significant (P>0.05). Furthermore, as expected with EMS mutagenesis, these mutations were distributed relatively evenly within the screened amplicons (Figure 2).

All the EMS-induced mutations were detected in single plants, except for the APRX gene, where the same mutation was found in two different plants. Only one induced stop codon mutation was identified for one of the five genes, APRX, also an splice mutant junction was identified in the ETR1 amino acid sequence.

Because of the high number of alleles identified, the possible impact of missense mutations on the function of the protein was assessed before systematic phenotyping of the mutant plants using SIFT (Sorting Intolerant From Tolerant). Out of the 32 missense mutations, a total of 14 mutations (44%) were predicted deleterious for the protein's activity (p<0.05): three and two missense mutations for ERS1 and ETR1 respectively, four for each of the two genes in the carotenoid synthesis and one missense mutation for APRX (Table S2).

### Genotype/phenotype association: characterization of the APRX mutants

Plant peroxidases are encoded by multigenic families and involved in several important physiological and development processes, although their varied functions are not clearly determined [30]. The appearance of nonsense mutants in these genes has not been described previously for this species so based on the genotype/phenotype relationship we decided to study the function of the only previously peroxidase studied in this species, APRX.

APRX is a globular protein of 330 aa whose structure is highly conserved between distant plant families. Along conserved residues, there are eight cysteine residues bounded by disulphide bridges and essential to stabilise the structure of the protein. The protein also shows thirteen alpha helices with an heme pocket and two distal Ca^2+^ binding sites disposed symmetrically in the center of the heme pocket. Throughout its structure of four exons and three introns we found nine families with mutations that affected diverse structural motifs.

For each APRX mutant family, the genotype/phenotype association was carried out using 18 M3 seeds that were germinated and genotyped by sequencing the APRX gene. No associated phenotype was observed in Cp-34 and Cp-773 families, which agrees with the position of the mutation in an intronic and non-coding region, respectively. Cp-675 and Cp-879 families, which showed a silent mutation, also did not show differences with the wild type.

Among the four mutant families that presented missense mutations, only the Cp-86 did not germinate, so it could not be evaluated. Cp-661 that presented an amino acid change at position R215 and Cp-359 and Cp-579 families with the same mutation at position S14F showed different phenotypes as dwarf plants or plants with deformed leaves. These phenotypes were caused by other mutations since, after genotyping of the lines, we could not associate these genotypes to a particular phenotype.

**Figure 3:**
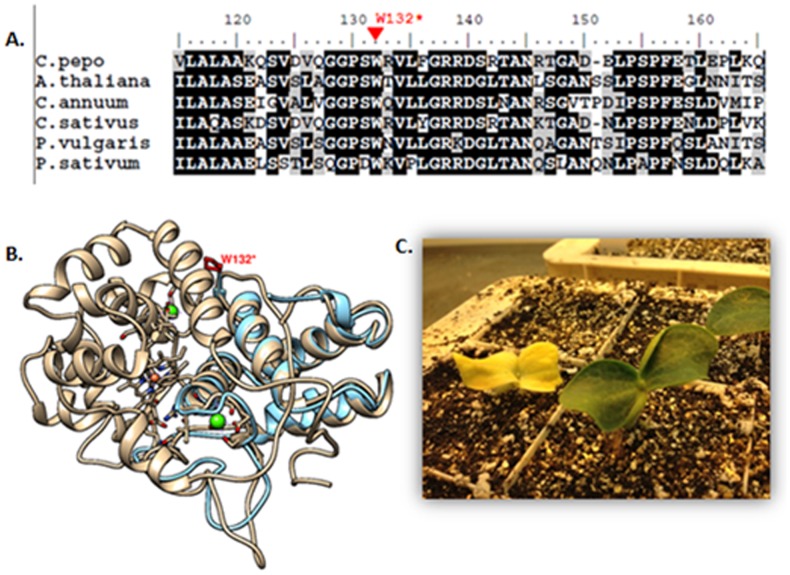
Sequence and structural analysis of APRX. (A) Amino acid alignment of *Cucurbita pepo* APRX and homologous proteins from *Arabidopsis thaliana* (AAM66044.1), *Capsicum annun* (AFU51540.1), *Cucumis sativus* (AAA33128.1), *Phaseolus vulgaris* (AAD37427.1) and *Pytum sativus* (BAD97438.1). Numbers above the alignment indicate the amino acid positions along the APRX protein. The EMS-induced stop mutation in W132 is shown above the alignment in red. (B) Superposition of the predicted 3D structure model of APRX WT protein indicated in grey and the mutant protein indicated in blue. The proximal heme pocket is represented in red and the two calcium ions in green. The position of the induced stop codon mutation indentified in Cp-161 family (W132*) is indicated in red. The APRX model was determined using the Geno3D server (http://geno3d-pbil.ibcp.fr). (C) Wild type and albino phenotypes observed in line Cp-161.

The induced stop codon mutation indentified in family Cp-161 (W132*) was the only mutation predicted to affect the protein function by SIFT. The nine germinated plants out of the 18 seeds sown were genotyped by sequence the APRX gene and six homozygous mutants, two heterozygous and one homozygous wild type were identified. The phenotype of seedling homozygous for the mutant allele was albino (Figure 3c). The remaining three M3 plants, corresponding to homozygous or heterozygous for the wild type allele, showed normal phenotypes.

Alignment of the homologous proteins showed that the W132* nonsense mutation occurred in the middle of a highly conserved protein motif. Based on the Geno3D derived structure of APRX and X-ray crystallography studies [31] (Figure 3b), the truncated mutant protein is predicted to miss helices A to D and the connected ability to bind heme and Ca^++^. Consistent with the mutant phenotype, the loss of more than half of the protein should be highly deleterious (Figure 3a).

## Discussion

The TILLING platform validated here is the first developed in *Cucurbita pepo*, probably because the low fertility that the mutants of this species have shown in previous attempts, even using different varieties [32], has hampered the perpetuation of the populations. The development of a method for selecting M1 lines with higher fertility used in the first steps of the generation of this current platform [20] was successful. Despite the mutagen's toxicity, the analysis of M2 and M3 lines performed in this study demonstrate that this TILLING platform consists of fertile lines with mutations affecting most phenological stages of development.

A primary objective in a mutagenesis project is to generate a saturated resource, where every *locus* is mutated and represented by multiple alleles. In the *C. pepo* TILLING platform more than one mutation was identified per analysed gene. We screened 768 M2 families to obtain a suitable allelic series which averages of 1 mutation per 133 Kb, so we can conclude that our mutant population is sufficiently saturated. Furthermore, by comparing our results with those reported in other TILLING projects with different plant species we can also affirm that our population is suitable for use in high-throughput mutation discovery. The density of mutations that we discovered in this assay appears to be much higher than the obtained for other cucurbit species such as *Cucumis melo* [13], [16] or *Cucumis sativus* [17] and if we compare this with other species, we find only five populations of diploid species with higher mutation densities: *Arabidopsis thaliana* [21], *Brassica rapa* [33], *Oryza sativa* [34], *Linum usitatissimum* [35] and a diploid wheat (*Triticum monococcum*) [36].

The high mutation ratio that we describe here comes from the analysis of one individual plant of each of the 768 M2 families. This mutation ratio is likely to be underestimated as M1 plants that are heterozygous for a new mutation will yield M2 families segregating 1∶2∶1 for the mutant homozygous, heterozygous and wild type homozygous. The sampling of only one plant per M2, even assuming good viability and fertility of the mutant allele, are likely to cause a bias in this segregation. Therefore, our next step will be to expand the population by generating DNA pools of at least four individuals per M2 family. This fact, maybe, could increase our mutation density. We also found that we had underreported mutations towards both ends of fragments and we attribute these losses primarily to a poor fluorescence signal [4].

A total of 58 mutations per 9 Mb screened were identified in 768 M2 plants and as expected for EMS mutagenesis, single nucleotide substitutions were identified both in coding and non-coding regions [4] and all the induced nucleotide changes by EMS were G/A and C/T substitutions (Table S2) [37].

All the EMS-induced mutations were detected in single families, except for one in the APRX gene, where the same change was found in two different families; this circumstance is expected and observed to occur 4% of the time in *Arabidopsis* based on random distribution of induced mutations, GC content of the genome and distribution of location of mutations discovered in fragments [4]. Finding one coincidence among 58 mutations is not significantly different from our expectations. Furthermore, finding that all 58 mutations are G/C-to-A/T transitions effectively rules out the possibility that they are naturally occurring polymorphisms and although G/C to A/T are the expected transitions, these changes are not always obtained [38]. This indicates that the EMS has had the predicted effect on the background and demonstrates no problems of crossing showing a population with a high homogeneity and inbreeding level.

Despite this higher mutation rate in the population, if we consider that about half of missense mutations are expected to be damaging to a typical protein [39], we expected to have about 16 deleterious mutations in the sequences studied. This fact agrees with the results based on SIFT algorithm which predicts deleterious missense mutations with ∼75% overall accuracy. According to SIFT, 44% out of the 32 missense mutations were predicted deleterious for the protein's activity (p<0.05) representing an extensive allelic series to address future structure-function studies in this species.

Phenotypic analyses confirm the phenotype/genotype correlation in APRX mutants. The anionic peroxidase analyzed [40], [41] is one of a broad family of Class III Peroxidases with up to 72 members identified in *Arabidopsis* genome [42]. The conserved structure and the low substrate specificity of these proteins have constituted a handicap to determine its specificity of function [28] because redundancy of function prevents phenotypes of overexpressed and antisense strategies. The functional analysis of this *C. pepo* peroxidase has been restricted to heterologous complementation in *A. thaliana*, where overexpression and silencing seems to indicate a role of APRX in auxin catabolism, but conclusive analysis is necessary to assess its specific function [28].

Evaluation of allelic series obtained by TILLING in *C. pepo*, which includes missense, nonsense and null alleles, constitutes an important resource to analyze the function of this gene. Some mutations were situated in significant structures of the protein. One in the signal peptide, and others in the alpha helix F, which conformation could change the position of one cysteine responsible of the structural conformation by covalent forces, however and according to SIFT, these mutations were tolerated and did not show any visual phenotype different from the wild type.

Finally, the truncated protein of line Cp-161, which showed a stop mutation identified at the conserved W132 residue, yielded albino seedlings that died in early stages when the mutant allele was homozygous, as was confirmed with the M3 genotyped plants. No differences from the wild type were observed when the mutant allele was heterozygous. Those results provide the first predicted nullimorphic mutation for APRX and a phenotype which is much more severe than that of previously reported mutants [28]. This phenotype could indicate the main role of this peroxidase at early stages of the plant's development and it could be related with the accumulation of hydrogen peroxide that becomes toxic and prevents the development of essential organelles of the cell such as chloroplasts. Although several studies are required to confirm this hypothesis, these mutants constitute a new source to obtain new insights of important and multifunctional proteins as peroxidases.

## Conclusions

The establishment of the EMS-mutagenized TILLING population described here represents an important advance in the generation of new genetic variation in *C. pepo*. The high mutation density obtained in this species makes it an attractive genetic system for the identification of new alleles that may be of value for crop improvement. Moreover, TILLING methodology becomes a feasible goal making possible the investigation of the role of key genes for this species bypassing the problems of other functional genomic tools. With the availability of this new resource we hope to fulfill the expectations of both, crop breeders and scientists, who are using this species as their model of study.

## Supporting Information

Table S1 **Primer pairs used to amplify the five genes tilled in** ***C. pepo*** **mutant population.** Asterisk indicate that the primer carried M13 tail and M13 universal primers, M13F700 (5′-CACGACGTTGTAAAACGAC-3′) and M13R800 (5′GGATAACATTTCACACAGG-3′), labelled at the 5′end with infra-red dyes IRD700 (forward primers) and IRD800 (reverse primers).
(DOC)

Table S2 **Allelic series of mutations identified in coding regions by TILLING.**
(DOC)
