# Supplementary material for: First TILLING Platform in Cucurbita pepo: A New Mutant Resource for Gene Function and Crop Improvement
Source: PLoS One. 2014 Nov 11;9(11):e112743. doi: 10.1371/journal.pone.0112743 (PMC4227871; doi:10.1371/journal.pone.0112743)
Supplement: Table S1 — Primer pairs used to amplify the five genes tilled in C. pepo mutant population. Asterisk indicate that the primer carried M13 tail and M13 universal primers, M13F700 (5′-CACGACGTTGTAAAACGAC-3′) and M13R800 (5′GGATAACATTTCACACAGG-3′), labelled at the 5′end with infra-red dyes IRD700 (forward primers) and IRD800 (reverse primers). (DOC) [file pone.0112743.s001.doc]

**Table S1. Primer pairs used to amplify the five genes tilled in *C. pepo* mutant population.** Asterisk indicate that the primer carried M13 tail and M13 universal primers, M13F700 (5′-CACGACGTTGTAAAACGAC-3′) and M13R800 (5′GGATAACATTTCACACAGG-3′), labelled at the 5′end with infra-red dyes IRD700 (forward primers) and IRD800 (reverse primers)

| **Gene** | | **Size (pb)** | **Forward** | **Reverse** |
| --- | --- | --- | --- | --- |
| PSY 1^st^ amplicon | | 759 | AAACAAAGTGGGGAGCTTCCTCCGT | CAAGCCAACAACAAACGAGGATAAAC |
|  |  |  | *CCAATTGGGTTTCTCGAT | *CAGTCGGCTCTATAACC |
| PSY 2^nd^ amplicon | | 758 | GGGCAATATATGGTAAGTGGTACTG | CACTCCTATCCGTGAATGTTTGAGC |
|  |  |  | *GGTTATAGAGCCGACTG | *CGATTATCGAGCTGAC |
| PSY 3^rd^ amplicon | | 763 | CATTTTTGGACTTTTCCTTTCGGGC | AGACCAGATTCAACGGTCACGAACC |
|  |  |  | *TTCCTCTCGAGGCTTT | *TGATTAGGGCCTCGAC |
| LCYb 1^st^ amplicon | | 1046 | TGTTTCTTGTTCAGTTCTTGAGGGT | AACAACTCTTTGAGGTAGAACGGGC |
|  |  |  | *GCTTATACTTGCTTGAG | *TCTTTCCTGGATATCG |
| LCYb 2^nd^ amplicon | | 764 | GATTGGAGGGATTCGCATCTGGATA | CGGGTATCGACCGAACATAATGAAG |
|  |  |  | *CGAATCGGATATTTCTCG | *TGACAGGTGTGAACTGAG |
| ERS1 1^st^ amplicon | | 1068 | GCTTAGCCTTCTTCAAGTTCATCCT | AATCACTGAGCTCTTACTTGACAGC |
|  |  |  | *GAGTCCTGTGATTGCATT | *CCTCAAGCATTAAGATCCT |
| ERS1 2^nd^ amplicon | | 1142 | CTAGCGATAGACTTGGGCGGTTAAA | AACAGAAAGAAGTTGGCTCAGACCA |
|  |  |  | *GCGGTATCAAGAATGTCA | *CGACAAACTGTTCGATG |
| ERS1 3^rd^ amplicon | | 471 | GATGAGACCGTTACAATGTAGCTAT | CCCATTTGGCATCCTTGTGTGTTCT |
|  |  |  | *CATCGAACAGTTTGTCG | *AGCGACGATCGAGATGTA |
| ETR1 1^st^ amplicon | | 978 | GCCGATCAGGTATTCATGAACCTGT | ACAAATTGCAAGCCCAAGACCACTG |
|  |  |  | *CATTCCTCCGTGCAATC | *TCGTGAACAACTTCGG |
| ETR1 2^nd^ amplicon | | 642 | GCCATTGTTGCAAAATCCGAAACCT | CTTTGGGATAGTGGCTGAAGAACAT |
|  |  |  | *CCAAGCGATAGTCAGTTT | *CCTACGTCTACTCGTTC |
| APRX 1^st^ amplicon | | 897 | TGGTCACTCATCCCGAATCCAAACT | TCCGGTGTGGTTGGATCGAAATTCA |
|  |  |  | *GTGTTACGTTTCGTTACC | *GAAGAACATGCATCTCGA |
| APRX 2^nd^ amplicon | | 629 | CTGGAGAGTTTTATTCGGAAGAAGA | GGGGTCTCCAACTGGGATGAACAT |
|  |  |  | *TCTTGTTGCTCCATCTGGT | *CACATGTTCCCTCCAGAA |
|  |  | | | |
