# Supplementary material for: First TILLING Platform in Cucurbita pepo: A New Mutant Resource for Gene Function and Crop Improvement
Source: PLoS One. 2014 Nov 11;9(11):e112743. doi: 10.1371/journal.pone.0112743 (PMC4227871; doi:10.1371/journal.pone.0112743)
Supplement: Table S2 — Allelic series of mutations identified in coding regions by TILLING. (DOC) [file pone.0112743.s002.doc]

**Table S2. Allelic series of mutations identified in coding regions by TILLING.**

| **Gene** | **Nucleacid transition** | **Amino acid substitution** | **SIFT prediction** | **Family name** |
| --- | --- | --- | --- | --- |
| **PSY** | C248T | S83L | 0.01 | CP-136 |
|  | G328A | D110N | 0.04 | CP-197 |
|  | G355A | G119R | 0.64 | CP-932 |
|  | C196T | P66S | 0.93 | CP-165 |
|  | C177T | H59H | 0.56 | CP-97 |
|  | G212A | S71N | 0.00 | CP-420 |
|  | G339A | P113P | 1.00 | CP-638 |
|  | G76A | V26I | 0.00 | CP-69 |
|  | C1128T | L232L | 1.00 | CP-595 |
|  | C773T | NON CODING | - | CP-909 |
|  | G738A | NON CODING | - | CP-853 |
|  | C2072T | NON CODING | - | CP-243 |
|  | C1762T | NON CODING | - | CP-815 |
|  | G1913A | G324R | 0.67 | CP-65 |
|  | G1895A | D318N | 0.42 | CP-396 |
|  | G2347A | NON CODING | - | CP-879 |
| **ERS1** | G283A | A95T | 0.01 | CP-94 |
|  | G732A | L244L | 0.62 | CP-523 |
|  | G393A | R131R | 1.00 | CP-506 |
|  | G640A | V214I | 1.00 | CP-739 |
|  | G1951A | A330T | 0.02 | CP-98 |
|  | G2102A | R380K | 0.61 | CP-449 |
|  | C2559T | H509Y | 0.53 | CP-897 |
|  | G1876A | A305T | 0.15 | CP-272 |
|  | C1865T | NON CODING | - | CP-84 |
|  | C2249T | NON CODING | - | CP-61 |
|  | C3120T | D595D | 0.64 | CP-58-3 |
|  | Before ATG | NON CODING | - | CP-116 |
|  | G3194A | R620K | 0.09 | CP-279 |
|  | C3200T | T622I | 0.01 | CP-17 |
|  | Before ATG | NON CODING | - | CP-40 |
|  | G3166A | D611N | 0.73 | CP-628 |
| **ETR1** | G1764A | D497N | 0.46 | CP-58 |
|  | G1673A | V466V | 1.00 | CP-55 |
|  | G1190A | R334K | 0.12 | CP-374 |
|  | C1474T | NON CODING | - | CP-921 |
|  | G1902A | G515R | 0.03 | CP-514 |
|  | C1978T | S540F | 0.62 | CP-795 |
|  | G2015A | SPLICE JUNCTION | 0.00 | CP-883 |
| **APRX** | G604A | W132* | 0.00 | CP-161 |
|  | G567A | NON CODING | - | CP-34 |
|  | C41T | S14F | 0.09 | CP-359 |
|  | C41T | S14F | 0.09 | CP-579 |
|  | Before ATG | NON CODING | - | CP-773 |
|  | G940A | R220Q | 1.00 | CP-86 |
|  | G926A | R215R | 0.20 | CP-675 |
|  | G925A | R215K | 0.14 | CP-661 |
|  | G929A | Q216Q | 0.34 | CP-879 |
| **LCYb** | G376A | A126T | 0.59 | CP-87 |
|  | C129T | V43V | 1.00 | CP-89 |
|  | Before ATG | NON CODING | - | CP-231 |
|  | G835A | A279T | 0.00 | CP-791 |
|  | G704A | G235E | 0.00 | CP-812 |
|  | G353A | G118E | 0.00 | CP-291 |
|  | G559A | V187I | 0.65 | CP-763 |
|  | G432A | T144T | 0.14 | CP-877 |
|  | C1013T | P338L | 0.00 | CP-525 |
|  | G896A | G299E | 0.13 | CP-435 |
